# Practical Aspects of Allogeneic Hematopoietic Cell Transplantation for Patients with Poor-Risk Chronic Lymphocytic Leukemia

**DOI:** 10.1100/tsw.2011.21

**Published:** 2011-01-18

**Authors:** Julio Delgado, Rafael F. Duarte

**Affiliations:** ^1^ Department of Hematology, Hospital de la Santa Creu i Sant Pau, Barcelona, Spain; ^2^ Department of Hematology, ICO-Hospital Duran i Reynals, Barcelona, Spain

**Keywords:** chronic lymphocytic leukemia, allogeneic hematopoietic cell transplantation

## Abstract

Allogeneic hematopoietic cell transplantation has become a viable option for younger patients with poor-risk chronic lymphocytic leukemia. The results obtained with either conventional or reduced-intensity conditioning regimens have been recently evaluated and compared with alternative nontransplant strategies. This manuscript deals with practical aspects of the procedure, including patient and donor selection, conditioning regimen, GVHD prophylaxis, disease monitoring, infectious and noninfectious complications, and timing of the procedure. Finally, we speculate on how we could improve the results obtained with the procedure and new advances currently in clinical trials.

### INTRODUCTION

Chronic lymphocytic leukemia (CLL) has been considered as a disease of the elderly and therefore not suitable for allogeneic hematopoietic cell transplantation (allo-HCT). However, there is a group of younger patients with poor-risk disease whose life expectancy is significantly reduced and for whom allo-HCT has been investigated as a potentially curative procedure. Moreover, the development of reduced-intensity conditioning (RIC) regimens and the greater availability of unrelated volunteer donors has increased the proportion of patients who might benefit from allo-HCT. Recently, the European Group for Blood and Marrow Transplantation (EBMT) concluded that allo-HCT was a reasonable therapeutic strategy for younger CLL patients who are refractory to fludarabine, those whose disease relapses within 2 years of intensive treatment, and those with 17p/p53 abnormalities requiring treatment[1].

In a recent review, the outcome of CLL patients undergoing allo-HCT was evaluated and compared with alternative therapeutic approaches[2]. In this manuscript, we discuss more practical issues regarding the actual procedure and how we could improve the results.

### WHO SHOULD BE REFERRED FOR ALLO-HCT?

The prognosis of patients with CLL who are refractory to fludarabine is dismal[3]. Alemtuzumab, a monoclonal antibody targeting CD52, has been licensed for fludarabine-refractory patients, but the overall response rate (ORR) is around 30–50% and the median overall survival (OS) is 16 months[4,5,6]. Furthermore, patients who are refractory to both fludarabine and alemtuzumab or, being fludarabine refractory, are unlikely to respond to alemtuzumab due to bulky lymphadenopathy, have a 23% ORR for the first salvage therapy and a median OS of 9 months[7]. Other promising alternatives, such as lenalidomide[8], flavopiridol[9], or rituximab plus high-dose methylprednisolone[10], have been tested in fludarabine-refractory patients, but none appears curative or provides acceptable long-term disease control.

The outcome of patients with a 17p deletion requiring therapy is also very poor[11]. Fludarabine combinations given at first line achieve a median progression-free survival (PFS) and OS around 12 and 24 months, respectively[12], while first-line alemtuzumab only achieved a median PFS of 11 months in a recent international randomized study[13]. Furthermore, the time to progression was only 8 months in patients with a 17p deletion who received alemtuzumab as salvage therapy[14].

Furthermore, the MD Anderson Cancer Center (MDACC) group recently evaluated the outcome of CLL patients relapsing after first-line fludarabine-cyclophosphamide-rituximab (FCR) therapy. Interestingly, patients relapsing within 36 months of FCR therapy had a significantly shorter median OS (12 months) compared to patients whose disease relapsed later (44 months, $p < 0.001$). Other factors significantly associated with a worse prognosis after frontline FCR were the presence of a 17p deletion, advanced Rai stage, and a high beta$_2$-microglobulin concentration[15].

In conclusion, CLL patients with poor-risk features, as identified by the EBMT group, have a dismal outcome with nontransplant strategies and should be offered an allo-HCT.

### WHEN SHOULD THE PROCEDURE TAKE PLACE?

Many CLL patients who should be referred for allo-HCT based on poor-risk features as described above will never proceed to transplantation. The Ohio State University group evaluated the outcome of 205 CLL patients who were referred for allo-HCT[16]. Only 34 (17%) of them had undergone transplantation at the time of the report, and the exact same number [34 (17%)] never proceeded to transplantation because the disease was unresponsive or the patient died from other complications. Other reasons for patients not proceeding to transplantation were: procedure not indicated or contraindicated (21%), patients' refusal (9%), insurance refusal (10%), and donor not available (3%). This survey highlights the difficulties of determining the appropriate timing for allo-HCT in CLL and the risks of delaying a transplant referral in patients with refractory disease.

In our opinion, if a CLL patient is eligible for allo-HCT and has a donor available, he or she should proceed to transplantation as soon as EBMT criteria are met. By definition, these patients have poor-risk disease and one should not wait too long if a reasonable response is obtained with salvage therapy, since the outcome following allo-HCT is significantly impaired once the disease has reached a state of complete unresponsiveness.

### MYELOABLATIVE OR RIC TRANSPLANTATION?

Traditionally, standard myeloablative allo-HCT has been associated with unacceptable toxicity and mortality in CLL patients. Early studies revealed a long-term nonrelapse mortality (NRM) and OS of 46% each[17]. More recent registry data comparing standard with RIC regimens showed a 23% 1-year NRM and 52% 5-year OS for the former, and a 22.5% NRM and 47% OS for the latter, but none of the differences were statistically significant[18].

Single-center studies have shown comparable results. The Dana-Farber Cancer Institute (DFCI) recently reviewed the outcome of 88 high-risk CLL patients undergoing a first allo-HCT from 1998 and 2007[19]. Sixty-two patients received a RIC regimen and 26 were conditioned with myeloablative chemoirradiation. At 3 years, there were no significant differences in OS between the groups (65 vs. 50% for patients receiving RIC and standard regimens, respectively), but NRM was lower in the RIC group (15 vs. 54%, $p < 0.001$). This lower NRM observed in the RIC group was counterbalanced by a higher 3-year relapse rate (30 vs. 8%, $p = 0.038$). These findings suggest that dose intensity may play a role in CLL control.

In contrast, a retrospective analysis performed at the Fred Hutchinson Cancer Research Center (FHCRC) revealed that CLL patients with comorbidities, whether young or old, had better long-term survival after RIC compared to conventional allo-HCT, whereas patients without comorbidities had comparable NRM, PFS, and OS. The same study also showed that the conditioning regimen only played a minor role in terms of disease control, with very similar relapse rates for both the myeloablative and RIC groups[20].

In conclusion, evidence that myeloablative conditioning is superior to RIC, even in younger or patients with fewer comorbidities, is lacking, although a potential role of conditioning intensity in patients with refractory/bulky disease or HLA-mismatched donor-recipient pairs cannot be excluded[21,22]. However, taking into account that median age at CLL diagnosis is 72 years and that conventional allo-HCT is rarely performed above 50 years of age, this discussion is often of no practical importance, as very few patients are ever considered candidates for conventional allo-HCT and RIC protocols may be chosen for the majority of patients.

### WHAT IS THE BEST RIC REGIMEN?

Several phase II studies have evaluated a variety of RIC regimens with different myeloablative and immunoablative potential (Table 1). The outcome of 82 patients conditioned with fludarabine and low-dose total-body irradiation (LDTBI) was recently updated by the FHCRC consortium[22]. At 5 years, the NRM and relapse rate were 23 and 38%, respectively, which translated into a 50% OS and 39% PFS. At the MDACC, 39 patients were conditioned with fludarabine, cyclophosphamide, and rituximab. At 4 years, NRM was 28%, current PFS was 44%, and OS was 48%[23].

Final results from the German CLL Study Group (GCLLSG) CLL3X trial have been recently published. Ninety patients received a variety of conditioning regimens (fludarabine plus cyclophosphamide, fludarabine plus busulfan, fludarabine plus LDTBI). At 4 years, the NRM, PFS, and OS were 23, 42, and 65%, respectively[24]. The British and Spanish Cooperative groups used a fairly similar, and slightly more intensive, RIC regimen. The Spanish group used fludarabine plus melphalan[25], whereas the U.K. group added alemtuzumab to the combination[26]. In a retrospective comparative study, NRM, PFS, and OS, with and without alemtuzumab, were 28 vs. 34% ($p = 0.735$), 39 vs. 47% ($p = 0.361$), and 65 vs. 57% ($p = 0.629$) at 3 years[27]. Patients receiving alemtuzumab had a lower incidence of extensive chronic graft-vs.-host disease (GVHD) (10 vs. 48%, $p = 0.03$).

The Cooperative German Transplant Study Group analyzed the clinical outcome of 30 CLL patients conditioned with fludarabine, busulfan, and thymoglobulin. At 2 years, OS and PFS were 72 and 67%, respectively. NRM was significantly higher in unrelated donor recipients compared to sibling donor recipients (28 vs. 0%, $p = 0.04$)[28]. Moreover, the DFCI evaluated the use of fludarabine plus busulfan. At 2 years, NRM was 17%, but the relapse rate was 48%. Median PFS was 10 months (34% at 2 years) and the 2-year OS was 54%[29].

A recently presented prospective randomized trial performed a head-to-head comparison of fludarabine plus LDTBI vs. fludarabine plus busulfan and ATG[30]. This trial recruited 139 patients with several hematological malignancies who had an HLA-identical sibling donor, but were ineligible for conventional myeloablative HCT. Patients conditioned with LDTBI had a significantly lower grade II–IV acute GVHD rate (26 vs. 51%, $p = 0.003$) and significantly shorter 1-year PFS (51 vs. 68%, $p = 0.048$),

**TABLE 1**
**Results of RIC allo-HCT for CLL**

| N | RIC Regimen | Age, Median (Range) | Chemo-refractory (%) | Prior auto-HCT (%) | Donor (% Related) | NRM | Grade II–IV aGVHD | Extensive cGVHD | Survival | Ref. |
|---|---|---|---|---|---|---|---|---|---|---|
| 82 | Flu-LDTBI | 56 (42–72) | 87 | 4 | 63 | 23% 5 years | 55% related; 66% unrelated | 49% related; 53% unrelated | 50% OS 5 years; 39% PFS | [22] |
| 39 | Flu-Cy-Ritux | 57 (34–70) | 28 | — | 90 | 28% 4 years | 45% | 58% | 48% OS 4 years; 44% CPFS | [23] |
| 90 | Flu-Cy based ± ATG | 53 ( 27–65) | 21 (47 FR) | 31 | 40 | 23% 4 years | 45% | 55% | 65% OS 4 years; 42% PFS | [24] |
| 41 | Flu-Mel-Alem | 52 (37–64) | 15 (43 FR) | 17 | 78 | 28% 3 years | 37% | 10% | 65% OS 3 years; 39% PFS | [27] |
| 21 | Flu-Mel | 54 (34–64) | 38 (29 FR) | 10 | 86 | 34% 3 years | 57% | 48% | 57% OS 3 years; 47% PFS | [27] |
| 30 | Flu-Bu-ATG | 50 (12–63) | 46 | 10 | 50 | 16% 2 years | 56% | 21% | 72% OS 2 years; 67% PFS | [28] |
| 43 | Flu-Bu | 53 (35–67) | 57 | 22 | 33 | 17% 2 years | 34% | 38% | 54% OS 2 years; 34% PFS | [29] |

Abbreviations: aGVHD, acute graft-vs.-host disease; Alem, alemtuzumab; ATG, thymoglobulin; Bu, busulfan; cGVHD, chronic graft-vs.-host disease; CPFS, current progression-free survival; Cy, cyclophosphamide; Flu, fludarabine; FR, fludarabine-refractory; HCT, hematopoietic cell transplantation; LDTBI, low-dose total-body irradiation; Mel, melphalan; NRM, nonrelapse mortality; OS, overall survival; PFS, progression-free survival; RIC, reduced-intensity conditioning; Ritux, rituximab.

but similar OS (74 vs. 75%). At 5 years, the relapse rate was also significantly higher for patients receiving LDTBI (50 vs. 28%, $p = 0.005$). Interestingly, patients conditioned with fludarabine plus LDTBI had a better quality of life and lower medical direct costs ($p < 0.001$).

In summary, long-term disease control can be obtained following a variety of RIC regimens in a substantial proportion of patients, particularly in those with chemosensitive and nonbulky disease at the time of transplantation. Unfortunately, every transplant group tends to use its “own” RIC regimen/s and firm recommendations on a particular RIC regimen cannot be made from the data available.

### WHAT IS THE BEST STRATEGY FOR GVHD PROPHYLAXIS?

The use of *in vivo* T-cell depletion (TCD) remains controversial in CLL patients undergoing RIC allo-HCT. This is usually achieved by giving alemtuzumab or thymoglobulin immediately before the stem cell infusion. Rates of extensive chronic GVHD can be as low as 10% when *in vivo* TCD is used, or as high as 59% when unmanipulated grafts are infused. Unfortunately, this reduced chronic GVHD rate is not necessarily associated with a lower NRM, and is counterbalanced by a delayed immune reconstitution, increased risk of severe infections, and increased relapse rate[27,31]. Furthermore, patients undergoing TCD transplants are more likely to require donor lymphocyte infusions, which are associated with higher rates of secondary chronic GVHD as suggested by a recent EBMT analysis[32].

Other research groups have focused on the immunosuppressive drugs given after transplantation. For instance, the DFCI group has pioneered the combination of tacrolimus and sirolimus as GVHD prophylaxis. Sirolimus is a potent immunosuppressive drug that has also been tested as lymphoma therapy due to its antineoplastic activity. A recent retrospective analysis revealed that patients receiving tacrolimus and sirolimus had a better OS compared to historical controls receiving tacrolimus plus methotrexate. Moreover, this beneficial effect was restricted to lymphoma/CLL patients undergoing RIC allo-HCT and was due to a reduced relapse rate (42 vs. 74% at 3 years, $p < 0.001$), not to a lower GVHD rate[33]. However, it must be kept in mind that sirolimus is associated with an increased risk of sinusoidal obstruction syndrome[34], thrombotic microangiopathy[35], and has many pharmacological interactions with drugs commonly used after transplantation, such as calcineurin inhibitors and antifungals.

### WHAT ABOUT THE DONOR?

Only a third of patients needing an allo-HCT have an HLA-identical sibling, meaning unrelated volunteer donors are required for a great proportion of patients. It is generally assumed that allo-HCT from HLA-matched unrelated donors achieves inferior results compared to HLA-identical siblings, but this has not been the case in CLL patients undergoing RIC allo-HCT[22,24,26]. Recently, Weisdorf et al. proposed a classification for unrelated donor-recipient pairs in order to better refine the concept of "matched unrelated donor". According to this classification, donor-recipient pairs are divided into well-matched, partially matched, and mismatched[36]. When this new classification was applied to the EBMT registry, Michallet et al. found that the outcome of CLL patients undergoing allo-HCT was determined by the degree of HLA matching[37]. Indeed, patients receiving stem cell grafts from well-matched unrelated donors had the same 5-year OS compared to identical siblings (59 vs. 55%, $p = 0.82$). In contrast, partially matched or mismatched donor-recipient pairs had a significantly worse OS (37%, $p = 0.005$) compared to any of the other subgroups. In conclusion, if a CLL patient requires an allo-HCT, well-matched unrelated donors provide similar results to identical siblings.

### WHAT ARE THE SPECIFIC PROBLEMS OF CLL PATIENTS DURING OR IMMEDIATELY AFTER ALLO-HCT?

#### High Graft Rejection Rates

CLL patients undergoing allo-HCT have remarkably high graft rejection rates. The incidence of graft rejection in patients undergoing RIC allo-HCT varies from 10 to 12% when grafts are T-cell depleted *in vivo* or *in vitro*[24,26], to 5 to 6% in the case of unmanipulated grafts[23,24]. A recent British-Spanish comparative study found a secondary rejection rate of 12 and 9% for patients conditioned with or without alemtuzumab, respectively[27].

A possible explanation for this phenomenon could be the significant marrow infiltration allowed in CLL patients at the time of transplantation, which would preclude HCT in patients with other malignancies. As such, at least three studies have shown an inverse correlation between the degree of marrow involvement and engraftment and/or outcome[29,24,38]. Perhaps a significant proportion of these patients have persistent CLL rather than a purely immunologic rejection as seen in patients with other diseases, such as aplastic anemia.

Moreover, Schetelig et al. explored whether the use of alemtuzumab as salvage therapy prior to transplantation had a significant impact on engraftment. Interestingly, they found that alemtuzumab levels >200 ng/ml on the day of the stem cell infusion were associated with a slower T-cell engraftment, although early withdrawal of immunosuppression and donor lymphocyte infusions (DLI) were able to solve the problem[39].

#### High Infection Rates

Infections are the most frequent cause of death in CLL patients that are refractory to fludarabine and/or alemtuzumab[3,7,40]. As a result, refractory CLL patients subjected to allo-HCT also have a particularly high incidence of infections compared to allo-HCT recipients with other lymphoid malignancies[41]. More than 60% of all nonrelapse deaths observed in the British-Spanish comparative study were caused by infections, most of them related to GVHD[27]. Other studies have shown similar rates of infectious nonrelapse deaths (30–66%), with a clear correlation between the risk of infections and the use of *in vivo* T-cell depletion or the presence of GVHD[22,23,25].

#### Modest Efficacy of Donor-Lymphocyte Infusions

There is strong circumstantial evidence of the existence of a graft-vs.-CLL (GVCLL) effect in patients undergoing RIC allo-HCT. Examples are lower relapse rates in patients with chronic GVHD[42], improved molecular responses upon withdrawal of immunosuppression[24], less relapses in recipients of unrelated donor grafts compared to HLA-matched siblings[38], higher relapse rates associated with the infusion of T-cell depleted grafts[24], and the modest efficacy of DLI[23,24,26,28].

In general, DLI are effective for reverting mixed chimerism, but less so in patients with progressive disease following allo-HCT[24,26,43]. Interestingly, in one study, the authors could detect CLL-reactive T-cell clones in patients who responded to DLI, but not in those with unresponsive disease[43]. In a similar study, patients achieving major antitumor response after allo-HCT developed $CD8^+$ and $CD4^+$ cells specific for CLL antigens, while patients with persistent disease despite GVHD did not have any CLL-reactive T cells[44]. In any case, response rates around 15% or less are expected after DLI in patients with relapsed disease[24,28,29].

Better results might be achieved with DLI given pre-emptively for persistent minimal residual disease (MRD). At the MDACC, 50% of patients with relapsed disease after HCT responded to DLI, although the interpretation of these results is complicated by the concomitant administration of rituximab[23].

#### Disease Relapse

The major cause of failure in CLL patients undergoing RIC allo-HCT is disease relapse. Early relapses usually happen in patients with refractory CLL that cannot be controlled by the conditioning regimen before the GVCLL effect can occur.

Unfortunately, late relapses have also been reported in the literature, even in patients with active GVHD. Several hypotheses have been proposed for this phenomenon, such as CLL clonal evolution, the development of tolerance[43], and the survival of tumor cells in "GVCLL sanctuary sites"[45]. Indeed, some of these relapses occurred in lymph nodes in the absence of bone marrow or peripheral blood involvement, even in patients with no MRD, further sustaining the existence of these sanctuary sites[46,47,48].

### HOW CAN WE IMPROVE THESE RESULTS?

#### Better Patient Selection

The EBMT guidelines identify a group of patients in whom available therapies are unlikely to achieve a prolonged disease-free survival and for whom allo-HCT is recommended[1]. In this respect, it is very important to perform fluorescent *in situ* hybridization tests for all CLL patients requiring therapy in order to identify those with a 17p deletion, and also to realize that patients whose disease relapses shortly after frontline FCR or FCR-like regimens should be considered for transplantation.

Beyond this recommendation, if we aim to improve the outcome and maximize the benefit of allo-HCT, we must incorporate into our decision-making algorithms additional patient- and disease-related factors. A FHCRC retrospective study showed that patient comorbidities were more important than CLL-

related variables for predicting PFS and OS[20]. Furthermore, an EBMT score designed many years ago to assess the risk of allo-HCT in patients with chronic myeloid leukemia[49] has been recently redefined to include patients with other hematological malignancies[50]. In the particular case of CLL patients, the EBMT score was also highly predictive of OS despite the fact that low scores were under-represented, as very few CLL patients were <40 years of age at the time of HCT and/or underwent HCT within 1 year of disease diagnosis[37]. Other studies have confirmed the negative impact of refractory CLL on NRM after allo-HCT[24,25,29].

Thus, it would appear that we could improve the outcome of allo-HCT in CLL if we were not only to exclude patients with unresponsive disease, but also to use the EBMT scoring systems and other comorbidity scores when deciding if a particular patient should proceed to transplantation or not (Table 2).

**TABLE 2**
**Allo-HCT for CLL in 2011 and Beyond: a Personal View**

| | Present | Future |
|---|---|---|
| Patient characteristics | • Younger than 70 (identical sibling) or 65 years old (unrelated donor)<br>• Low comorbidity index | • EBMT risk score 1–4 (?) |
| Disease characteristics | • Refractory to fludarabine, OR<br>• Early relapse following intensive therapy (e.g., FCR), OR<br>• 17p deletion or p53 mutation requiring therapy | • Refractory to bendamustine (?) |
| Therapy prior to allo-HCT | • Any therapy able to achieve the best possible response<br>• Avoid alemtuzumab 1 month before transplantation | |
| Conditioning regimen | • Usually reduced intensity due to old age and comorbidities<br>• Consider myeloablative in case of refractory or bulky disease | • Addition of anti-CD20 monoclonal antibodies (?) |
| Donor | • Identical sibling, OR<br>• Well-matched unrelated donor | |
| GVHD prophylaxis | • *In vivo* T-cell depletion generally NOT recommended, at least in identical siblings | • Sirolimus as an immunosuppressive AND anti-CLL agent (?) |
| Post-HCT monitoring | • Regular MRD studies in peripheral blood and bone marrow<br>• Regular CT scans<br>• Strict infectious disease surveillance (e.g., CMV, EBV in case of T-cell depletion, galactomannan, etc.) and prophylaxis (e.g., antivirals, antibacterials, antifungals, immunoglobulin infusions, etc.) | |
| Maintenance therapy post-HCT | • Not standard yet<br>• May be difficult to implement | • Monoclonal antibodies (?), lenalidomide (?) |

### Better Timing

Physicians taking care of younger CLL patients fulfilling EBMT criteria for allo-HCT should not hesitate to refer them for transplantation. A donor search should be initiated and the procedure should take place as soon as a reasonable response is achieved with chemoimmunotherapy. If the decision is not taken promptly, the disease may become unresponsive and the procedure is much less effective in this situation.

### Modulation of the GVCLL Effect

Rituximab, an anti-CD20 monoclonal antibody, may facilitate disease control when given concomitantly with RIC allo-HCT or DLI[23]. This may be achieved by direct cytotoxicity and modulation of the GVCLL effect. Interestingly, rituximab has been shown to promote the cross-presentation of tumor-derived peptides by antigen-presenting cells, thus enhancing the formation of cytotoxic T-cell clones and the GVCLL effect[51]. Indeed, the addition of rituximab or other anti-CD20 antibodies to RIC regimens has been already tested[23,52] and is the focus of several ongoing clinical trials.

### Maintenance Therapy

Maintenance therapy with monoclonal antibodies has become very popular in patients with follicular lymphoma and other indolent lymphoproliferative disorders, such as CLL[53,54]. Moreover, lenalidomide is an immunomodulatory agent with proven activity in poor-risk CLL[8,55] that can be administered orally and is currently being tested as maintenance therapy in the same patient subgroups. Both agents could be explored in order to reduce the risk of relapse after allo-HCT.

### Post-Transplant Monitoring of CLL Patients Undergoing allo-HCT

Apart from standard post-transplant monitoring tests (e.g., donor-recipient chimerism tests, CMV monitoring, etc.), several studies have stressed the value of MRD surveillance in CLL patients undergoing allo-HCT[45,47,56,57]. There is no doubt that patients achieving a negative MRD status have a lower relapse risk compared to those with positive MRD after allo-HCT[45,47,56,57], and MRD studies are therefore generally recommended in all CLL patients undergoing allo-HCT. Unfortunately, we still do not know if MRD-based pre-emptive strategies (immunosuppression withdrawal and/or DLI) improve the results of the procedure[24].

On the other hand, CT scans have been recently incorporated into the assessment of disease response to therapy, at least in the context of clinical trials[58]. As previously mentioned, the disease could progress in the lymph nodes in the absence of bone marrow and/or peripheral blood involvement[46,47,48], further emphasizing the important role of imaging studies in the early detection of disease relapse after allo-HCT.

Finally, it is of critical importance to follow these patients closely and strictly observe anti-infective prophylactic measures, including prompt immunoglobulin infusions in case of severe hypogammaglobulinemia in view of the severe immune deficiency and high infectious nonrelapse mortality of these patients[41].

## CONCLUSIONS

Allo-HCT is a very powerful tool in the management of poor-risk CLL patients. Patients who can expect a significant reduction of life expectancy with conventional therapy should be identified promptly and referred for allo-HCT before the disease becomes unresponsive. Due to old age, frequent marrow involvement, and pre-existing immune suppression, these patients are prone to a number of CLL- and transplant-related complications, and should be managed with great care. Novel transplant strategies, such as MRD-based immunomodulation, newer drugs for GVHD prophylaxis, the addition of monoclonal antibodies to the conditioning regimen, or post-transplant maintenance strategies, could improve the results, but should be tested in the context of clinical trials.

### ACKNOWLEDGMENTS

We are indebted to Dr. Peter Dreger for all his wonderful ideas and suggestions.

### CONFLICT OF INTEREST DISCLOSURES

J.D. has received lecturing and consulting fees from Bayer Schering Pharma, Roche, and GlaxoSmithKline. R.F.D. has received consulting fees from Bayer Schering Pharma.
